# Injustice to Doctors

**Published:** 1890-11

**Authors:** 


### EDITORIAL DEPARTMENT.

F. E. DANIEL, M. D., EDITOR AND PUBLISHER.

THIS JOURNAL, by unanimous vote of the members, was made the Official Organ of the Austin District Medical Society.

COLLABORATORS:

*Dr. R. M. Swearingen, Austin.*
*Prof. B. E. Hadra, M. D., Galveston.*
*Prof. Geo. Cupples, M. D., San Antonio.*
*Dr. T. C. Osborn, Cleburne, Texas.*
*Dr E. J. Doering, Chicago.*
*Dr. E. J. Beall, Fort Worth.*
*Dr Odo Betz, Germany.*
*Prof. W. B. Rogers, M. D., Memphis.*
*Dr. J. W. McLaughlin, Austin.*
*Dr. T. J. Tyner, Austin.*
*Prof. J. F. Y. Paine, M. D., Galveston.*
*Dr. R. H. L. Bibb, Mexico.*
*Dr. T. J. Bennett. Austin.*
*Dr. Bat Smith, Wharton.*
*Dr. E. Meierhof, New York.*
*L. H. Luce, M. D., West Tisbury, Mass.*

### INJUSTICE TO DOCTORS.

On several occasions heretofore the JOURNAL has inveighed against the gross injustice done physicians in compelling them to serve as "experts" in State cases without compensation; or, if paid at all, they are paid as witnesses,—and about $1.50 a day.

Upon what pretext they are paid as witnesses, it is hard to understand; they are *witnesses* in no sense. A witness testifies to *facts;* an expert testifies as to accepted theories, or hypotheses,—for medicine is not an exact science, nothing, we may say, is positively known; a witness states what he *knows*; an expert is *compelled* under duress to state what he *thinks;* to give his opinion. How *unlike* a witness is he? Were a witness to say "I think" or "I believe," he would be incontinently "sat down upon" and told that no one wants his opinion; yet a physician, who in all probability knows nothing whatever of the case in hand, is summoned as an "expert," called a witness, and paid as a witness, and yet is compelled by fear or threat of fine or imprisonment, to state—*not* what he knows, but what he thinks! How inconsistent and how unjust!

A case has recently occurred here at Austin, in which precisely the above took place,—except the small matter of pay,—the gentleman did not receive even the paltry $1.50 per day and yet paid his own expenses to and fro. But we anticipate.

One of the most eminent and able of the Austin physicians — a man whose practice is no doubt worth $25 a day—at a low estimate—was summoned before the District Court at Waxahachie, in Ellis county,—some 175 miles from Austin. He failed to obey, and a sheriff was sent for him with an attachment. Taken thus by force from his home and his practice, he was compelled to pay his own expenses to Waxahachie—his hotel and other bills and his return fare. He was put on the stand and gave "expert" testimony in a case, the merits of which he knew no more about than did the man in the moon. He would have been paid his witness fees (?) at the end of three days, but for an *informality*, *a lapsus* of the red tape,—a technical error made by the clerk in the wording of the document. So, after rendering this important service to the State, or to the litigants, as the case may be, he returned to Austin a poorer, a sadder—if not a wiser man. Outside of his expenses—say $25—he lost three days time, which may safely be put down at $75. Thus, this private citizen was virtually *robbed* of $100. He was made to "stand and deliver"—and had no discretion. Comment is unnecessary.

A physician's knowledge represents his stock in trade, his capital. The highwayman has as much right to despoil the traveler of his personal effects at the muzzle of a six shooter, as a court has to force a physician to give expert testimony without compensation, and under pressure,—fear,—the certain knowledge of fine and imprisonment. Is there no remedy for this lamentable state of affairs?

In the same category,—as far as the injustice of the thing goes—is our correspondent from Brownwood in this issue, (see Dr. Johnson's letter: "A Texas soldier attempts to regulate fees," etc.) The Doctor faithfully served the State, perhaps saved the lives of its soldiers,—paid for, out of his private means, the medicines furnished them—and now—through a defect in the laws of the State he has no redress, but must suffer the injustice in silence. Is there any other class of citizens who are thus imposed upon? We opine not; in every trade, calling and profession, except that of medicine, one has redress at law. The mechanic has a lien on the building he constructs, which takes precedence over all other claims; as to lawyers,—*they* get their pay in advance,—shrewd fellows—and are not in the count; the merchant has ample protection at law, no one can go in and take *his* goods without pay. The Doctor only—the patient and long suffering,--the faithful, charitable, benevolent, kind-hearted Doctor, who, rather than see and know that a human being is suffering, will go to his relief if he never gets paid—he alone of all the State's citizens is thus cruelly and shamefully imposed upon. Is there no remedy? Let our State Medical Association—or its Committee on Medical Legislation, see to it that at the next session of the legislature a bill is passed to provide for the payment of expert testimony, and that something is done to meet such exigencies as are related in Dr. Johnson's letter.

